# Project GIVE: using a virtual genetics service platform to reduce health inequities and improve access to genomic care in an underserved region of Texas

**DOI:** 10.1186/s11689-024-09560-x

**Published:** 2024-09-09

**Authors:** Blake Vuocolo, Roberta Sierra, Daniel Brooks, Christopher Holder, Lauren Urbanski, Keila Rodriguez, Jose David Gamez, Surya Narayan Mulukutla, Ana Hernandez, Alberto Allegre, Humberto Hidalgo, Sarah Rodriguez, Sandy Magallan, Jeremy Gibson, Juan Carlos Bernini, Melanie Watson, Robert Nelson, Lizbeth Mellin-Sanchez, Nancy Garcia, Lori Berry, Hongzheng Dai, Claudia Soler-Alfonso, Kent Carter, Brendan Lee, Seema R. Lalani

**Affiliations:** 1 Department of Molecular and Human Genetics, Baylor College of Medicine, One Baylor Plaza, R806, Houston, TX 77030 USA; 2 Primary and Community Care, University of Texas Rio Grande Valley, Harlingen, TX 78550 USA; 3 DHR Health Diabetes and Endocrinology Institute, Edinburg, TX 78539 USA; 4 Department of Otolaryngology, University of Texas Rio Grande Valley, Harlingen, TX 78550 USA; 5 Vannie E. Cook Jr. Children’s Cancer and Hematology Clinic, McAllen, TX 78503 USA; 6 Milestones Therapeutic Associates, McAllen, TX 78501 USA; 7 Department of Pediatrics, University of Florida, Jacksonville, FL 32207 USA; 8 Rio Grande Valley, Easterseals, TX 78505 USA; 9 Baylor Genetics Laboratories, Houston, TX 77030 USA; 10 Texas Children’s Hospital, Houston, TX 77030 USA

**Keywords:** Virtual access to genomics, Consultagene, Genome sequencing, Medically underserved population

## Abstract

**Background:**

The utilization of genomic information to improve health outcomes is progressively becoming more common in clinical practice. Nonetheless, disparities persist in accessing genetic services among ethnic minorities, individuals with low socioeconomic status, and other vulnerable populations. The Rio Grande Valley (RGV) at the Texas-Mexico border is predominantly Hispanic/Latino with a high poverty rate and very limited access to genetic services. Funded by the National Center for Advancing Translational Sciences, Project GIVE (Genetic Inclusion by Virtual Evaluation) was launched in 2022 to reduce the time to diagnosis and increase provider knowledge of genomics in this region, with the goal of improving pediatric health outcomes. We describe our experience of establishing a virtual pediatric genomic service in this region to expeditiously identify, recruit, and evaluate pediatric patients with undiagnosed diseases.

**Methods:**

We have utilized an innovative electronic health record (EHR) agnostic virtual telehealth and educational platform called Consultagene to receive referrals from healthcare providers in the RGV. Using this portal, genetic services, including virtual evaluation and genome sequencing (GS), are being delivered to children with rare diseases. The study has also integrated effective methods to involve and educate community providers through in-person meetings and Continuing Professional Education (CPE) events.

**Results:**

The recruitment efforts have proven highly successful with the utilization of Consultagene in this medically underserved region. The project’s ongoing engagement efforts with local healthcare providers have resulted in progressively more referrals to the study over time, thus improving inclusion and access to genomic care in the RGV. Additionally, the curated CPE content has been well received by healthcare providers in the region.

**Conclusions:**

Project GIVE study has allowed advanced genetic evaluation and delivery of GS through the virtual Consultagene portal, effectively circumventing the recognized socioeconomic and logistical barriers to accessing genetic services within this border community.

**Supplementary Information:**

The online version contains supplementary material available at 10.1186/s11689-024-09560-x.

## Background

Rare genetic diseases are known to disproportionately affect children, causing early childhood death or chronic physical and/or neurodevelopmental challenges. Diagnosing children in a timely manner is crucial for providing appropriate disease-specific intervention, counseling families about recurrence risks, and addressing the psychosocial and financial challenges that are known to be associated with diagnostic odysseys [1]. However, within the United States healthcare system, low-income communities and ethnic minorities often do not have equitable access to genetic services [2, 3]. Multiple studies have shown that Hispanic/Latino individuals living in the United States are less likely to get genetic testing compared to non-Hispanic/Latino groups [4–6]. 

There currently exists a substantial gap in the access to genomic healthcare for the community along the southernmost tip of the Texas-Mexico border called the Rio Grande Valley (RGV), which has a population of about 1.4 million residents [7]. Over 94% of the population in the RGV identifies as Hispanic/Latino; approximately 30% of individuals under the age of 65 years are uninsured; and 30-40% of children live in poverty [8, 9]. The RGV has four counties (Starr, Cameron, Hidalgo and Willacy), three of which are amongst the Texas counties with the highest concentrations of colonias, defined as residential areas that lack basic living necessities such as potable water and sanitation infrastructure [10]. All four counties in the RGV are designated medically underserved areas by the Health Resources and Service Administration (HRSA), indicating an inadequate amount of primary care services to address the health needs of the population [11, 12]. 

Access to genomic care is not only restricted by low socioeconomic status of the residents, but also by limited familiarity of the local healthcare professionals with genetic disorders. Previous studies have indicated that primary care providers in a Federally Qualified Health Center generally found clinical advantages in providing genetic testing to their patients. However, a significant number of them lacked training in genetics and did not integrate genetic service into their practices [13]. Even when children with rare diseases are eventually identified for assessment, the limited availability of highly trained and board-certified full-time pediatric geneticists in this region necessitates families to travel significant distances to large pediatric centers for comprehensive genomic care. Families facing financial, logistical, or social challenges are unable to make the journey, resulting in inability to access these vital services and receive timely genetic diagnoses. The interwoven socioeconomic and healthcare system barriers in this region result in prolonged diagnostic odysseys, which in turn can lead to preventable health declines, missed opportunities for participation in clinical trials, and recurrence of frequently devastating diseases within families. There is an urgent need to implement alternate modes of delivery of care that could be readily integrated into the workflow of healthcare providers in the RGV to improve the lives of children with genetic disorders.

Project GIVE (**G**enetic **I**nclusion by **V**irtual **E**valuation), an NIH-funded research study, has been designed to provide state-of-the art virtual genetic evaluation and whole genome sequencing (GS) for children with rare diseases in the RGV using Consultagene, an academically-developed virtual genetics service platform, with the goal of reducing their time to diagnosis (TTD). Furthermore, efforts are aimed at improving the genomic competency of local healthcare providers through educational events to expedite genetics referrals for children with suspected genetic diseases. The trial is registered in Clinicaltrials.gov (Identifier NCT05318222). The study is conducted at the University of Texas Rio Grande Valley (UTRGV) and Baylor College of Medicine (BCM). Here, we describe the processes and insights gained from the study design and our engagement with the healthcare providers in the RGV to advance pediatric genomic care. We also outline our experience of using a virtual genomics evaluation platform to deliver both remote evaluation and GS for children with rare diseases living in this region.

## Methods

### Utilization of a telehealth platform

Between 2016 and 2018, a HIPAA-compliant EHR-agnostic genetics tele-engagement platform called Consultagene was developed at BCM to improve access to genomic care and education for individuals with limited access to a genetics specialist [14]. This virtual platform offers comprehensive services, including patient scheduling, medical document sharing, interpretation of genetic test results, tele-genetic counseling, and educational videos for patients and healthcare providers, which are available in multiple languages including Spanish. The platform’s features are modular, which allows it to adapt to the unique needs of both clinical and research settings. Healthcare providers and patients themselves can submit a referral request through Consultagene for a patient to be scheduled and seen by one of BCM’s clinical genetic counselors via video conference. Referrers are also able to submit a request for a “peer-to-peer” consultation, whereby they can consult with a BCM provider through the platform and obtain genetics advice on any given case. Since 2019, Consultagene has clinically engaged patients requiring counseling for prenatal testing, preconception, and in vitro fertilization considerations, as well as specialized consultations related to cancer genetics and neurodegenerative diseases such as Huntington’s disease and Alzheimer’s disease. Project GIVE presents a novel use of this technology in a research setting that caters to the pediatric rare disease population coupled with the delivery of GS.

### Inclusion and exclusion criteria

Children who are 0–18 years of age are eligible to participate in Project GIVE if they reside in the RGV and have a suspected underlying genetic etiology for their medical presentation. Families must primarily speak English or Spanish to participate. Participants are not eligible if they already have a genetic diagnosis that explains their symptoms. In this phase of the study, Project GIVE assesses children within an outpatient setting. Neonates with suspected genetic disorders are directed to Consultagene for evaluation following their discharge from the hospital.

### Selection of study site, participant recruitment, and application review

In collaboration with UTRGV, the UT Health RGV Pediatric Specialty Clinic in Edinburg, Texas was strategically selected as the primary study site because of its ease of access for families. Additionally, there is a diverse range of pediatric subspecialists in this single facility, including a neurologist, a developmental pediatrician, and a pulmonologist, which presented adequate opportunities for patient referrals. The Consultagene kiosk was set up in this clinic for families to engage with the Project GIVE genetics team located in Houston.

**Fig. 1:**
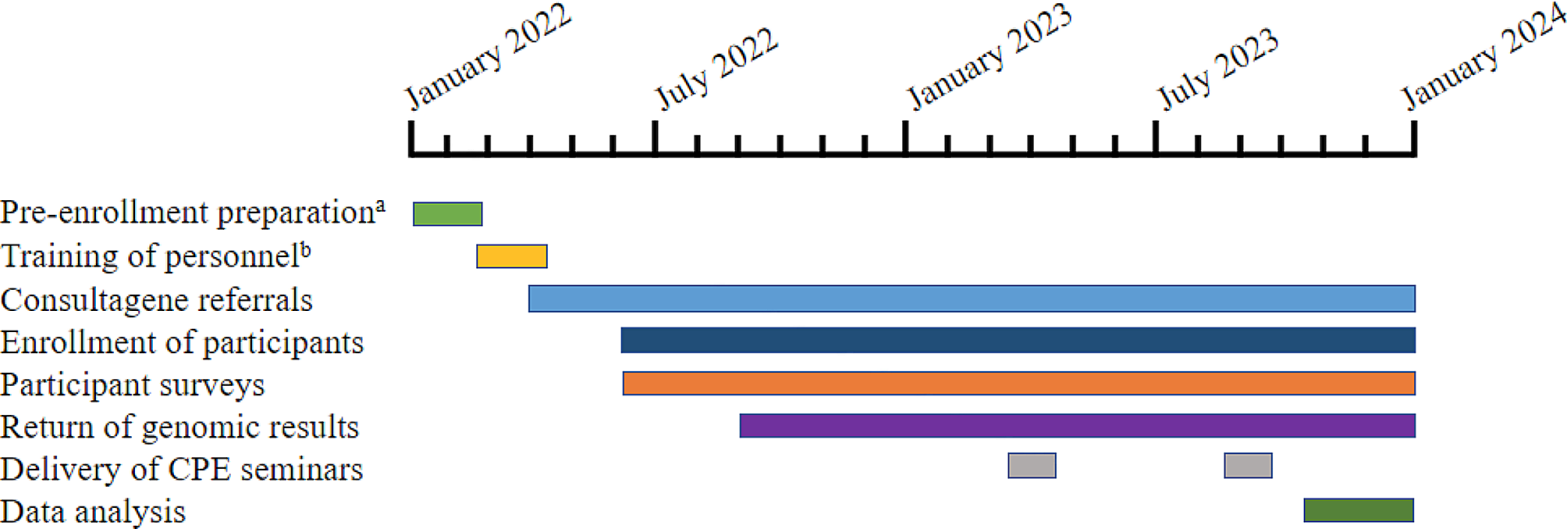
Study timeline. ^a^ Pre-enrollment preparation included development of the IRB protocol, identification of study site at the UTRGV pediatric multi-specialty clinic in Edinburg, TX and installation of the Consultagene kiosk. ^b^ Personnel includes research coordinator and UTRGV pediatricians who participate in Project GIVE clinical meetings. We began enrolling patients shortly after receiving our first referrals. Return of genomic results appointments were scheduled once results were returned from the lab. Outreach to community providers (including genetics providers visits to the RGV and visits to clinics by our research coordinator) has been ongoing throughout the study period

Project GIVE has been designed to recruit approximately 100 pediatric participants from the RGV between February 2022-January 2024 (Fig. 1) for genetic evaluation and GS. Prioritizing inclusion and access to care, the referrer base for this study has been extended to a wider network of healthcare professionals in this region, including physicians, nurses, nurse practitioners, medical assistants, early childhood intervention (ECI) specialists, as well as physical, occupational, and speech therapists taking care of children with complex medical needs in rehabilitation centers. The UTRGV pediatricians, along with the project’s local research coordinator (R.S.), who has a background in social work and prior experience at a local geneticist’s office, work together to identify community healthcare professionals who could potentially refer study participants. The bilingual research coordinator regularly visits clinics to proactively distribute study flyers and discuss the benefits of the study for children with complex medical conditions. Additionally, the team of clinical geneticists and the genetic counselor based in Houston conduct multiple visits to this region to engage in face-to-face meetings with local clinicians.

The healthcare providers send in a referral through Consultagene requesting “Peer-to-Peer consultation”. After accessing the portal, the referrers are prompted to upload pertinent clinical information including available electronic health records for their patients. The Project GIVE clinical team, consisting of geneticists and a genetic counselor at BCM, the research coordinator, and the UTRGV pediatricians, convenes remotely on a weekly basis to review the referrals and ascertain those most likely to benefit from genetic evaluation.

Additionally, the study team has established a collaborative partnership with the Texas Department of State Health Services’ Texas Birth Defects Registry (TBDR), which plays a pivotal role in identifying pregnancies and infants with birth defects in Texas [15]. The registry team conducts routine visits to pediatric hospitals, birthing centers, and midwife facilities throughout Texas to identify affected individuals. The TBDR team conducts systematic reviews of medical records, and both the International Classification of Diseases (ICD) codes for birth defects and text descriptions are detailed for each patient. Information for patients diagnosed with birth defects between 2015 and 2020 in the RGV is accessible to the Project GIVE study team as approved by the Institutional Review Board of the Texas Department of State Health Services. Utilizing our established provider engagement process, our team facilitates a Consultagene referral for the patient through the patient’s pediatrician.

### Delivering virtual genetic evaluation with GS and longitudinal follow-up via Consultagene

**Fig. 2:**
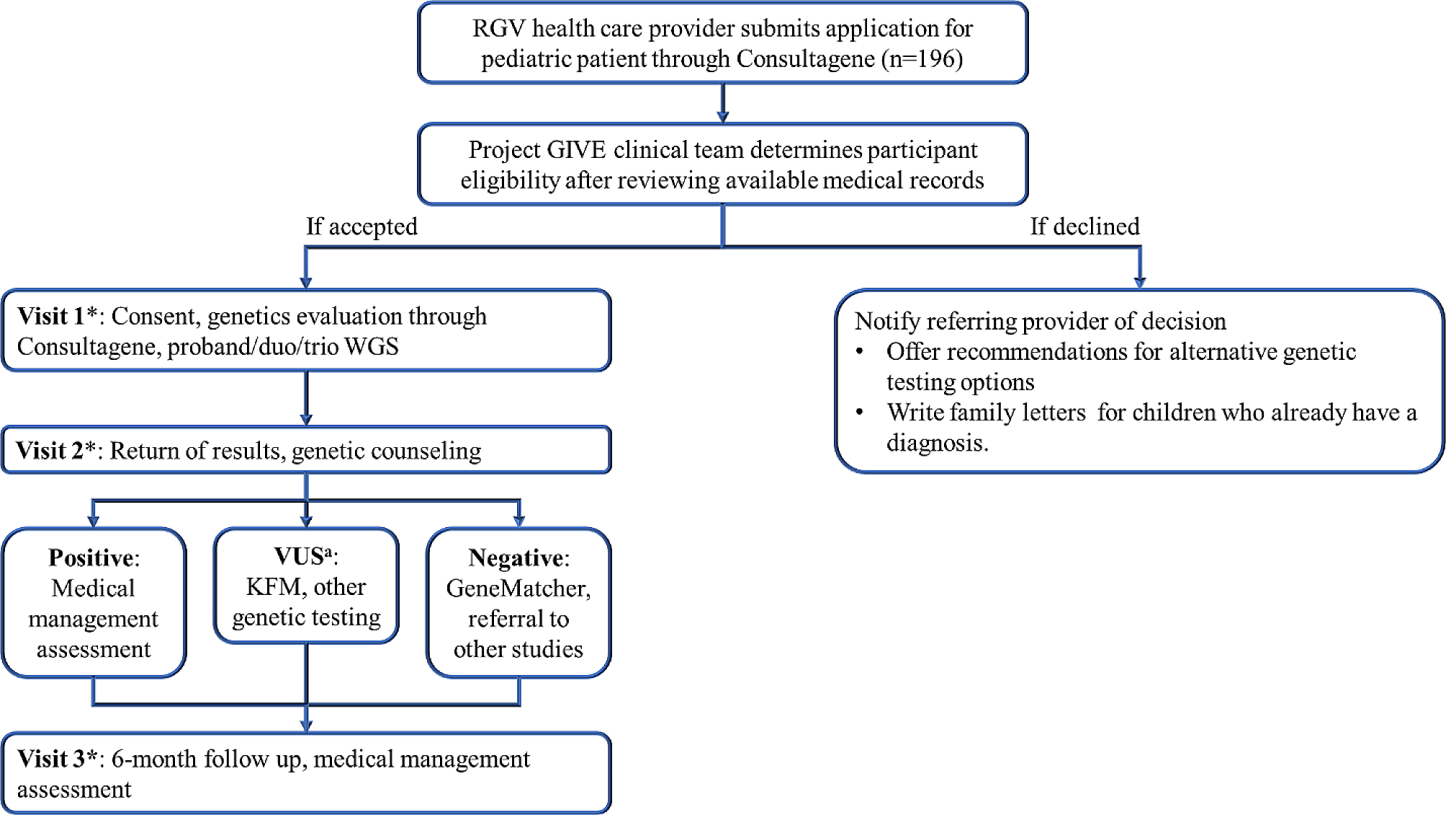
Project GIVE evaluation process. Participants participate in 3 study visits over one year. KFM = known familial mutation. * indicates timepoints in which survey data is collected from families (including demographic information, perceptions of genetics, and experiences receiving genetic test results). ^a^ KFM of affected/unaffected siblings or family members or other genetic testing (ex. RNA sequencing) may be recommended by the study team to help resolve a VUS

Figure 2 illustrates a participant’s journey from referral to the final study visit. Upon acceptance into the study, participating families undergo a structured three-visit protocol over one year. At the initial encounter, “Visit 1”, the research coordinator meets with the pediatric participant and their family at the study site for the informed consent process. After being consented into the study, families then access two educational videos in their preferred language that are integrated in the Consultagene platform: (1) *Basics of Genetics*, and (2) *What to Expect at a Genetics Clinic Visit.* Due to the lower genetic health literacy levels of many of the study participants and their limited exposure to genetics, these videos provide relevant context to the patients regarding the research study. Families complete a five-question survey, including three 5-point Likert scale questions about their perspectives on the eduational videos and two knowledge questions related to the content covered in the videos. Following this, the clinical geneticists and genetic counselor, located in Houston, conduct an in-depth remote evaluation and physical exam of the pediatric participant through Consultagene’s videoconferencing platform. The virtual assessment is facilitated by the research coordinator present at the study site. Anthropometric measurements, including weight, height, and head circumference, are gathered for all participants by a medical assistant at the study site.

The research coordinator is also responsible for collection of buccal samples for proband, duo, or trio GS and coordinating transfer to Baylor Genetics, a Clinical Laboratory Improvement Amendments (CLIA)-certified laboratory in Houston, TX, where a clinical GS report is generated. Families can opt-in to receiving the ACMG medically actionable secondary findings during the consenting process [16]. The genetics evaluation and GS studies are both carried out as part of the research study and are not billed through families’ insurance.

When the clinical team receives the GS results in ∼ 4–8 weeks, a videoconference return of results (ROR) call is scheduled with the families for “Visit 2”. Positive cases undergo an in-depth review of the diagnosis and medical management, with accompanying informational letters crafted for both providers and patients, in their preferred language. The Project GIVE’s clinical team facilitates follow up appointment referrals in the RGV whenever possible. In cases where it is clinically necessary, patients are directed to specialty clinics at Texas Children’s Hospital (TCH) in Houston, which is approximately 350 miles away from the study site. When a variant of uncertain significance is ascertained, familial testing is undertaken to interpret the genomic results. In the event of a negative GS result, further exploration of any research findings detailed on the GS report takes place, including clinical labs/imaging, and/or GeneMatcher submissions. Additionally, selected patients are referred to additional research studies like the Undiagnosed Diseases Network.

“Visit 3”, occurring approximately 6 months post-disclosure, serves as a follow-up to collect additional medical information about the child and review the GS results again. A detailed summary of each visit is meticulously documented and promptly shared with the referring providers through the Consultagene portal to facilitate effective communication.

### Enhancing genomic competency of healthcare professionals

A critical component of Project GIVE involves training and educating referrers to ensure expeditious patient referrals to genetic services. In 2023, we developed and delivered two Continuing Professional Education (CPE) seminars designed to cater to the unique needs of non-genetics providers in this region. The content for both events focused on the importance of genetic testing and recognition of rare diseases in pediatric patients. When designing the curriculum for these events, our goal was to help local healthcare professionals develop greater confidence in referring patients to genetics, understanding different genetic testing methodologies, and interpreting genomic results at some level to address families’ basic inquiries. Additionally, we introduced conference attendees to Face2Gene [17], a machine-assisted technology for identifying patients eligible for genetics referral. Attendees also received updates on the regional impact of Project GIVE. At the September 2023 CPE event, attendees completed a pre-CPE survey that collected provider and clinic information and asked questions about how comfortable providers were with ordering, interpreting, and explaining different genetic tests. They also completed a post-CPE survey that allowed them to answer the same questions about comfortability with genetic tests. Responses for the pre- and post-CPE surveys were linked using a study ID that was randomly assigned to the attendees. Survey data were analyzed using descriptive statistics, and responses for the comfortability questions were paired to assess whether there were changes in responses at the two time points.

### Expected study outcomes

The primary outcome measure of Project GIVE is TTD of rare diseases, with the starting point designated as the date when a healthcare provider initially referred the patient to Consultagene. Patients are longitudinally followed for a duration of up to one year post-referral. Event time is computed in months as the time from referral to the date of ROR.

Additionally, we have incorporated harmonized surveys developed by the Clincial Sequencing Evidence-Generating Research (CSER) consortium that were designed to study the effectiveness of integrating genomic sequencing into the clinical care of ethnically diverse and medically underserved individuals [18–21]. These measures are suitable for participants with low literacy levels and are fully translated into the Spanish language. Survey data are collected to characterize sociodemographic variables, literacy, understanding, perceived utility of sequencing, and satisfaction with the ROR process. Surveys are administered to families at each of the three study visits. As the study design of Project GIVE is the specific emphasis of this paper, the TTD analysis and the survey data regarding participants’ experiences will be explored in a forthcoming publication.

## Results

### Referrals and evaluations

**Fig. 3:**
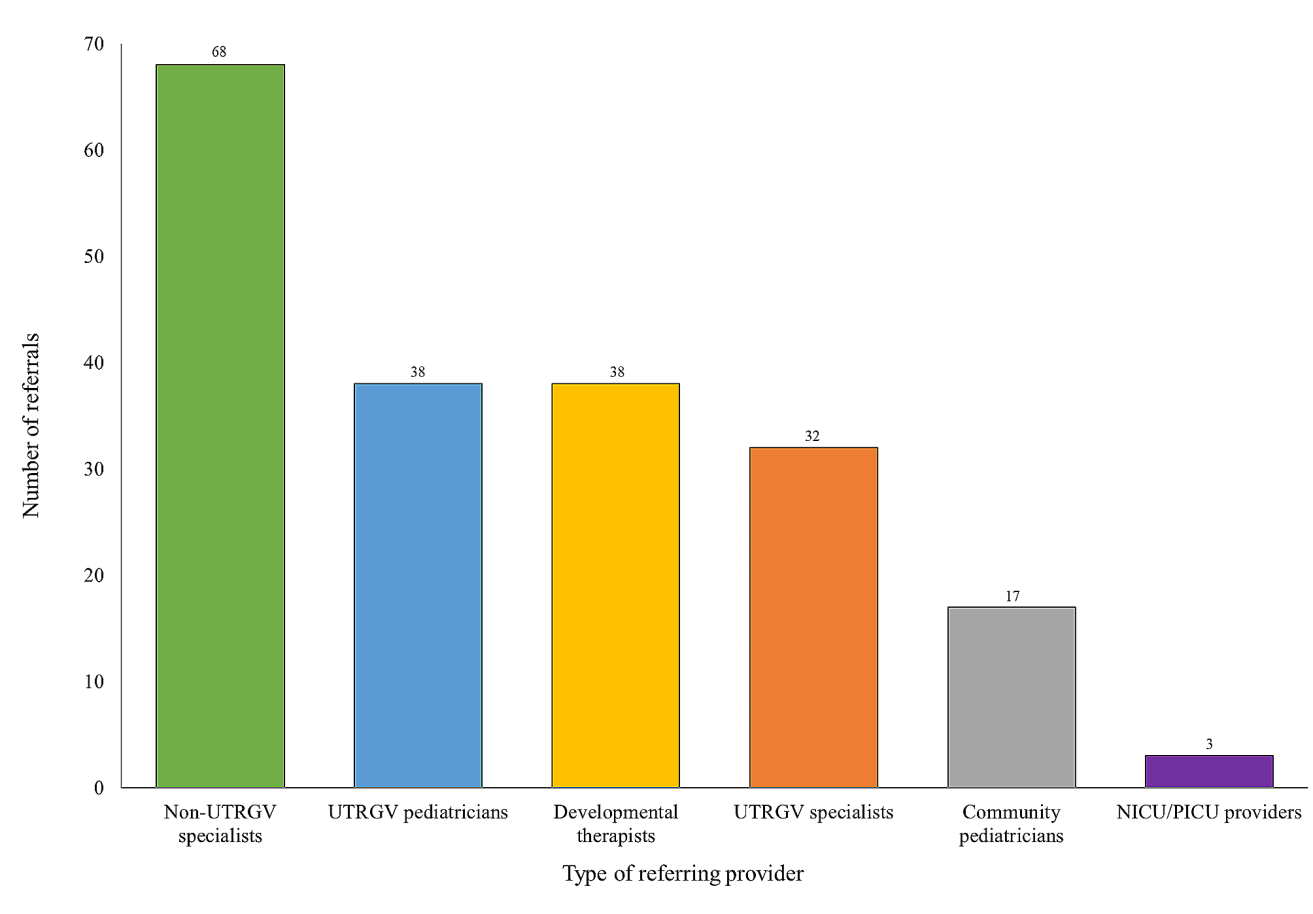
Referrals to Consultagene by healthcare provider designation (*n* = 196). Specialists include pulmonologists, psychiatrists, developmental pediatricians, endocrinologists, oncologists, and audiologists

Between February 2022 and January 2024, 196 pediatric patients (ages 0–18 years) with suspected undiagnosed rare diseases were referred through the Consultagene portal by healthcare professionals in the RGV. Notably, 18 referrals (9.2%) were from private-practice community pediatricians and 39 referrals (19.8%) were from developmental and rehabilitation therapists. The bulk of referrals came from pediatricians, endocrinologists, developmental pediatricians, pulmonologists, neonatologists, oncologists, and audiologists in the region (Fig. 3). Approximately 80% of the accepted study participants predominantly presented with a neurodevelopmental phenotype – including intellectual disability, seizures, developmental delay, autism spectrum disorder, behavioral/mental health concerns, and neuromuscular disease – often with other organ systems affected as well.

### Participant surveys

About 98% of enrolled families identified as Hispanic/Latino, and 40% of participants preferred communicating in Spanish with their healthcare providers. About half of enrolled families reported an annual household income of <$20,000 in the last year. The 5-question survey regarding the Consultagene video content was completed by 9/60 enrolled families. Results from this survey data suggest that families are overall satisfied with these educational videos (Supplemental Fig. 1).

### Clinician engagement and CPE events

**Fig. 4:**
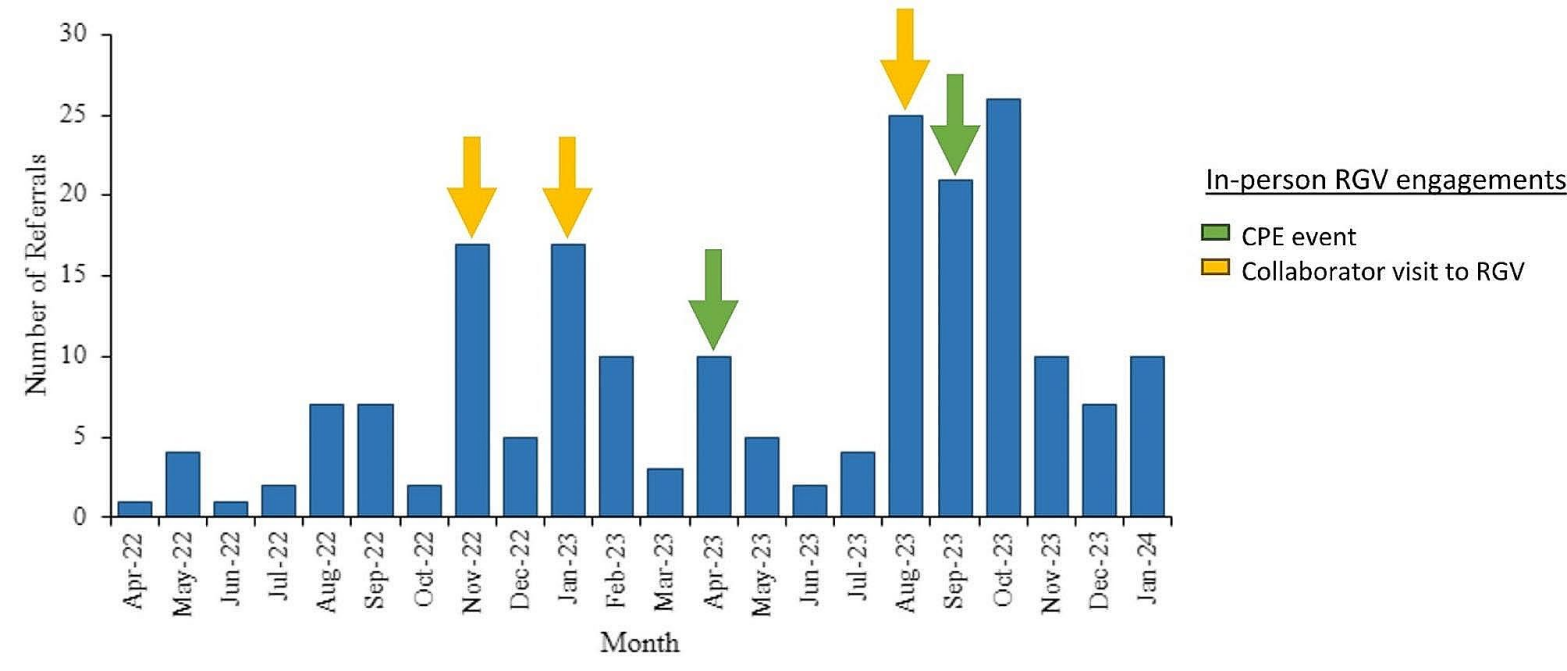
Number of referrals to Consultagene by month (*n* = 196). Arrows indicate months in which Houston Project GIVE team traveled to the RGV for in-person engagement with healthcare providers. CPE events (green arrows) were held on 4/15/23 and 9/23/23. Collaborator visits (yellow arrows) included presentations to local pediatricians, specialty groups, and hospital departments

We strengthened collaborations with community healthcare providers in the RGV by engaging in multiple in-person meetings with local pediatricians and subspecialists. These in-person engagements have cultivated strong connections with healthcare providers in the region, leading to a surge of Consultagene referrals around times of our visits, as well as an overall incremental increase in referrals to our study over time (Fig. 4).

Additionally, two CPE seminars were held on April 15th, 2023, and September 23rd, 2023. In total, 32 pediatricians, specialists, developmental therapists, and social workers attended these meetings (19 attendees at the April 2023 event; 13 attendees at the September 2023 event). Data collected at the September 2023 CPE event indicated growing confidence of the healthcare professionals in approaching children with genetic disorders after attending the CPE event (Supplemental Fig. 2). We continue to actively engage with attendees of the meetings and seek their input in selecting future curricula, fostering a collaborative approach.

### Evolution of the study protocol

Project GIVE was originally designed as a peer-to-peer study, and we envisioned participation of the referrers for all three study visits throughout the Project GIVE journey. However, once we began recruiting, we recognized that there were several logistical barriers to scheduling patients around the busy providers’ schedules. We subsequently transitioned to a more traditional telemedicine set-up whereby the study geneticist, genetic counselor, and research coordinator met with the families independently of the referring providers.

At the outset, our team planned to establish a framework for assessing referrals, expecting each submission to contain detailed medical records and previous genetic evaluations. Over time, it became increasingly apparent that accessing comprehensive medical records for referred participants was challenging. These children routinely receive care from multiple specialists across different hospital systems that employ various EHR systems. Frequently, supporting ancillary investigations, such as brain MRIs, EEG records, X-rays, echocardiograms, and any previously-completed genetic tests, are inaccessible to our team. In a significant number of referrals, only a concise clinical note regarding the medical concerns is submitted via the Consultagene portal. To address potential barriers posed by limited available medical records for these families, our clinical team has revised the acceptance criteria to cater to the majority of referrals. Through our experience, we have learned that even a concise narrative, when combined with information about the involvement of multiple specialists in the patient’s care, is often adequate for identifying those who would benefit from GS. We have found it necessary to amend our original study framework and prioritize a genotype-driven approach to expedite diagnoses in this frequently under-phenotyped population.

While our efforts have aimed to maximize enrollment opportunities, approximately 20% of referred patients have not been accepted. In some cases, we discovered that the child already had a genetic diagnosis that the clinical team/family were unaware of, and in other cases, the clinical indication did not warrant a comprehensive sequencing work-up; for example, the child may have exhibited a milder phenotype, or the phenotype may have suggested a genetic diagnosis that could be identified through more targeted testing. We have made continued efforts to provide quality care for all patients who have been referred to Project GIVE, regardless of their acceptance into the study. For individuals referred who had a previously identified diagnosis, we provide a detailed letter to both the provider and the family that explains the diagnosis, inheritance pattern, and necessary follow-up care. For patients who would benefit from alternative genetic testing options, we work with the referring providers to facilitate the ordering of the most suitable test. Consequently, most patients referred to us will undergo an appropriate genetics workup.

## Discussion

The integration of genomic information to enhance health outcomes is becoming more prevalent in clinical practice across the country. Nevertheless, disparities in accessing genetic services among ethnic minorities and individuals with low socioeconomic status have resulted in the marginalization of the most vulnerable populations. Consultagene has emerged as a transformative solution, positioned to contribute significantly towards reducing TTD in underserved communities with limited resources. Given the scarcity of local access to clinical geneticists in the region, this advanced referral tool has played a pivotal role in streamlining patient pathways and enabling genetic evaluation of children with rare diseases. It represents one of the first systematic initiatives to integrate virtual health delivery with GS, particularly focused on a medically underserved pediatric population.

A previous study from a Hidalgo county specialty clinic in the RGV during COVID-19 found that only 15.6% of their patients opted to be seen via telemedicine rather than in-person, and while 92.4% of the community identified as Hispanic/Latino, only 69% of individuals who opted to use telemedicine were Hispanic/Latino. The authors speculated that this difference could be attributed to limited access to technology, technological literacy, or cultural perceptions of telemedicine [22]. Our decision to integrate Consultagene into a local RGV clinic allowed us to circumvent some of these proposed challenges.

Within our research team, we are fortunate to have a bilingual study coordinator native to the RGV who has an intimate understanding of the region’s culture and challenges. She has emerged as a trusted member of our group, fostering strong connections with both providers and participants, especially those who prefer communication in Spanish. With training in social work, she assists families in accessing additional support beyond the study’s genetic services, such as identifying school and mental health resources, housing assistance programs, and local specialists. In essence, her role has evolved into a patient navigator role commonly implemented in cancer settings to reduce barriers to care for patients from underserved backgrounds [23, 24]. Her comprehensive care plays a crucial role in the success of our virtual tele-genetics program. As such, we advocate for the integration of a patient navigator into similar virtual genetics programs in other underserved regions.

A recognized challenge in initiating a genomic study in an underrepresented region involves establishing trust among medical providers and community members. The UTRGV clinicians, who are already trusted medical professionals in the community, have continually utilized their networks in the region to help ascertain children most likely to most benefit from Project GIVE. Furthermore, our efforts to establish a wide referrer base – including physicians, nurse practitioners, medical assistants, and ECI specialists – has significantly expedited the referral of patients requiring urgent evaluation to Project GIVE. This strategic inclusion has enhanced the accessibility and efficiency of our services.

### Virtual evaluation limitations

While the virtual setup of our study has expanded access to genomic care, conducting a remote physical examination comes with its limitations. The information gathered may often be insufficient, potentially leading to the oversight of pertinent features crucial for phenotyping and informing GS. To address the constraints of telemedicine examinations, our research coordinator collects photographs after informed consent and electronically transfers the images to the study team for detailed assessment. We emphasize the importance of having an in-person coordinator to help facilitate the genetics evaluation.

## Future directions

In the months ahead, we will continue to recruit pediatric patients with undiagnosed diseases living in the RGV. Concurrently, we are committed to analyzing the CSER survey data to gain a deeper understanding of parental experiences with and perceptions of genetic testing. Furthermore, we are in the process of concluding a qualitative study that explores the barriers and facilitators to accessing comprehensive medical care and social/educational services for children in the RGV with rare diseases. The combination of quantitative and qualitative data from our cohort will highlight the experiences of families in the RGV with children facing complex, undiagnosed diseases and will provide insight into the longitudinal experiences that families may face after receiving a diagnosis. This information is crucial for informing best practices to improve genomic health equity along the Texas-Mexico border.

When considering the sustainability of these genetics services in the region beyond Project GIVE, it is important to consider whether a true peer-to-peer consultation model would increase access to care for children with undiagnosed diseases in this region while still providing patients with quality genetics services. Considering that GS is currently fully funded by our study’s grant, it’s imperative to acknowledge the potential impact of insurance status on families’ future access to testing.

## Conclusions

In summary, Project GIVE has successfully increased access to genomic care in a medically underserved, predominantly Hispanic/Latino population by implementing a virtual telehealth platform and offering GS to pediatric patients with rare diseases. We strongly believe that these initiatives are readily applicable and replicable in other regions with limited healthcare resources.

## Electronic supplementary material

Below is the link to the electronic supplementary material.

**Supplementary Material 1: Supplemental Figure 1** | Participant responses on the Consultagene post-video survey (*n* = 9). A five-question survey was administered to participants after they watched the *Basics of Genetics* and *What to Expect at a Genetics Clinic Visit* videos in the Consultagene portal. Three questions (A, B, C) assessed participants’ perceptions of the videos, and two true/false questions (D, E) assessed participants’ understanding of the material covered in the videos. Only six participants completed the final question (E)

**Supplementary Material 2: Supplemental Figure 2** | RGV providers’ pre- and post-CME survey responses (*n* = 10, paired). Attendees of the CPE events were asked to complete questions pertaining to their comfortability with different aspects of genetics before (left column graphs) and after (right column graphs) the CPE content was presented

## Data Availability

The datasets used and/or analyzed during the current study are available from the corresponding author on reasonable request.

## Abbreviations

CPE: Continuous Professional Education
CSER: Clinical Sequencing Evidence-Generating Research
ECI: Early Childhood Intervention
GS: Whole Genome sequencing
Project GIVE: Genetic Inclusion by Virtual Evaluation
RGV: Rio Grande Valley
ROR: Return of results
TBDR: Texas Birth Defects Registry
TTD: Time to Diagnosis
UTRGV: University of Texas Rio Grande Valley
