# Supplementary material for: Project GIVE: using a virtual genetics service platform to reduce health inequities and improve access to genomic care in an underserved region of Texas
Source: J Neurodev Disord. 2024 Sep 9;16:52. doi: 10.1186/s11689-024-09560-x (PMC11382520; doi:10.1186/s11689-024-09560-x)
Supplement: Supplementary file 1 — Supplementary Material 1: Supplemental Figure 1 | Participant responses on the Consultagene post-video survey (n = 9). A five-question survey was administered to participants after they watched the Basics of Genetics and What to Expect at a Genetics Clinic Visit videos in the Consultagene portal. Three questions (A, B, C) assessed participants’ perceptions of the videos, and two true/false questions (D, E) assessed participants’ understanding of the material covered in the videos. Only six participants completed the final question (E) [file 11689_2024_9560_MOESM1_ESM.docx]

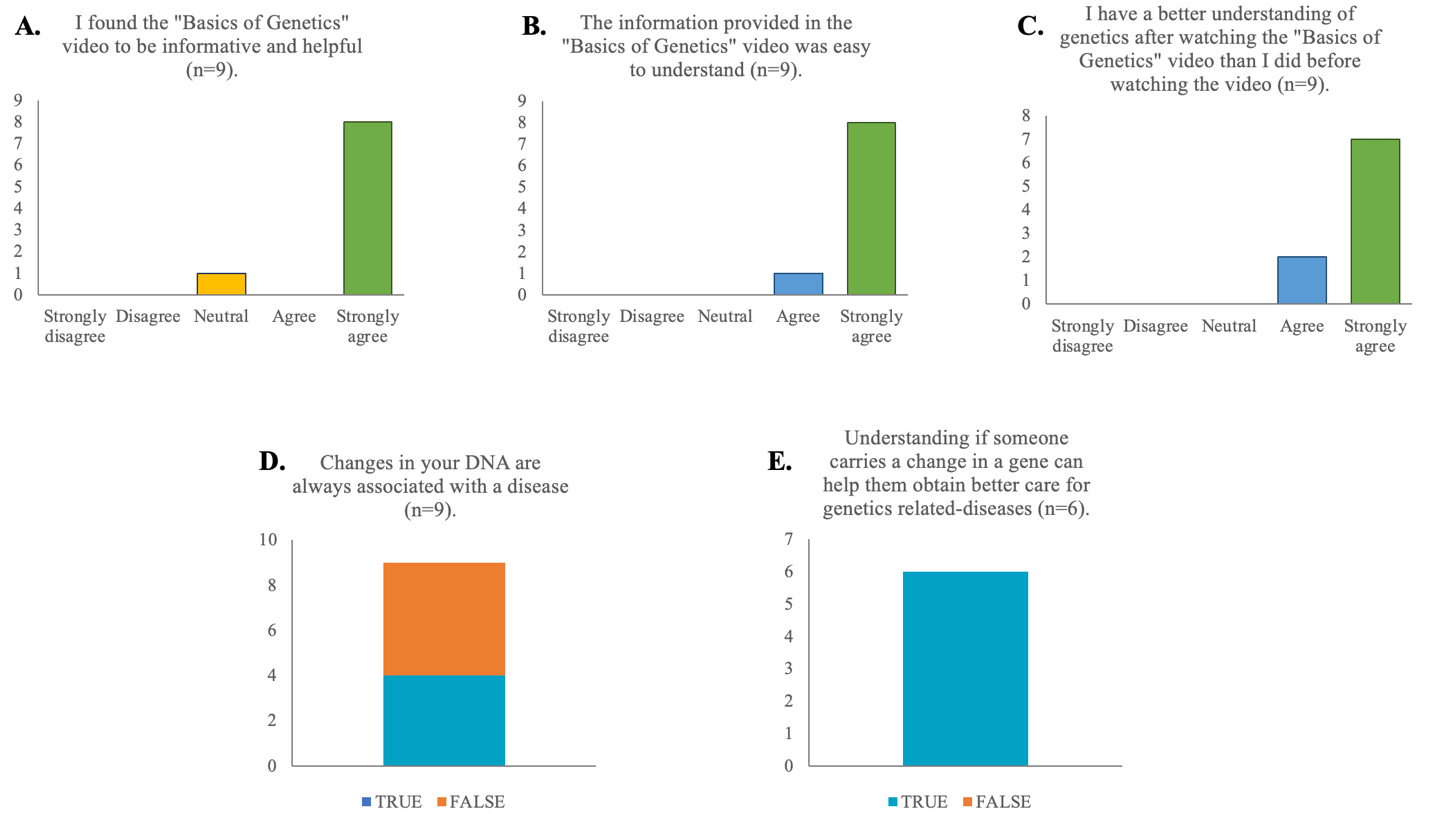
**Supplemental Figure 1 | Participant responses on the Consultagene post-video survey (n=9)**. A five-question survey was administered to participants after they watched the *Basics of Genetics* and *What to Expect at a Genetics Clinic Visit* videos in the Consultagene portal. Three questions (A, B, C) assessed participants’ perceptions of the videos, and two true/false questions (D, E) assessed participants’ understanding of the material covered in the videos. Only six participants completed the final question (E).
