# Supplementary material for: Project GIVE: using a virtual genetics service platform to reduce health inequities and improve access to genomic care in an underserved region of Texas
Source: J Neurodev Disord. 2024 Sep 9;16:52. doi: 10.1186/s11689-024-09560-x (PMC11382520; doi:10.1186/s11689-024-09560-x)
Supplement: Supplementary file 2 — Supplementary Material 2: Supplemental Figure 2 | RGV providers’ pre- and post-CME survey responses (n = 10, paired). Attendees of the CPE events were asked to complete questions pertaining to their comfortability with different aspects of genetics before (left column graphs) and after (right column graphs) the CPE content was presented [file 11689_2024_9560_MOESM2_ESM.docx]

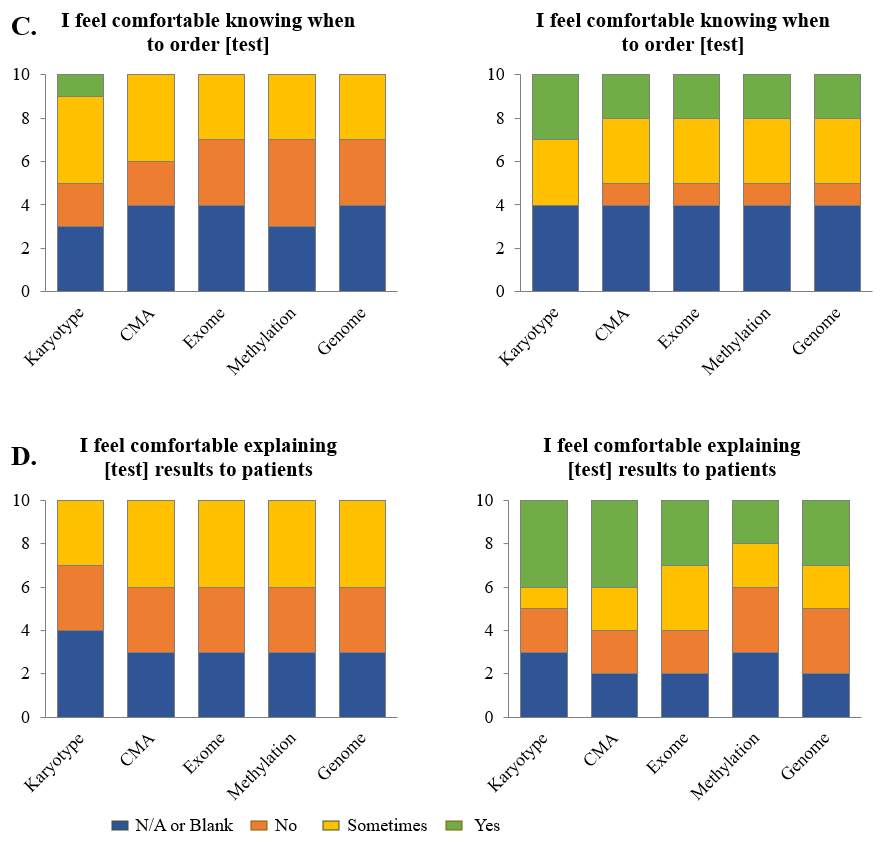

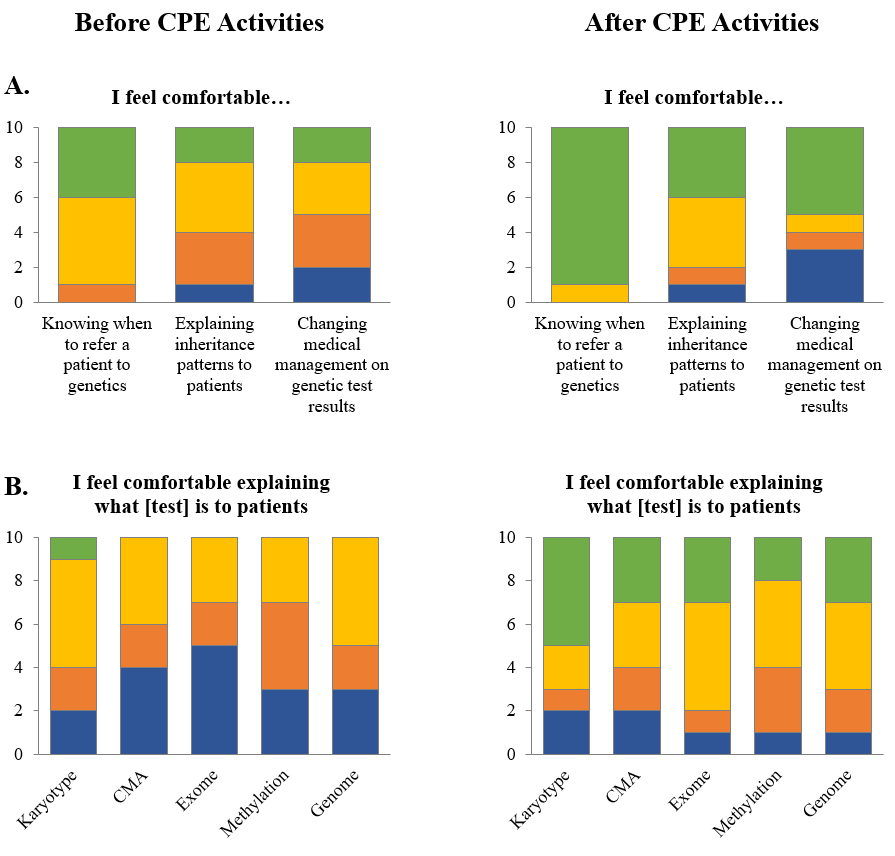


**Supplemental Figure 2 | September 2023 RGV attendees’ pre- and post-CPE survey responses (n=10, paired).** Attendees of the September 2023 CPE event (n=13) were asked to complete questions pertaining to their comfortability with different aspects of genetics before (left column graphs) and after (right column graphs) the CPE content was presented. CPE content included information regarding when to refer a patient to genetics, detailed information on different causes of genetic disease and genetic tests that can be ordered, and information on how to review and interpret different genetic test results. Three attendees did not complete the post-survey and therefore were not included.
